# Combined Effects of Extreme Climatic Events and Elevation on Nutritional Quality and Herbivory of Alpine Plants

**DOI:** 10.1371/journal.pone.0093881

**Published:** 2014-04-04

**Authors:** Annette Leingärtner, Bernhard Hoiss, Jochen Krauss, Ingolf Steffan-Dewenter

**Affiliations:** Department of Animal Ecology and Tropical Biology, Biocentre, University of Würzburg, Würzburg, Germany; University of Marburg, Germany

## Abstract

Climatic extreme events can cause the shift or disruption of plant-insect interactions due to altered plant quality, e.g. leaf carbon to nitrogen ratios, and phenology. However, the response of plant-herbivore interactions to extreme events and climatic gradients has been rarely studied, although climatic extremes will increase in frequency and intensity in the future and insect herbivores represent a highly diverse and functionally important group. We set up a replicated climate change experiment along elevational gradients in the German Alps to study the responses of three plant guilds and their herbivory by insects to extreme events (extreme drought, advanced and delayed snowmelt) versus control plots under different climatic conditions on 15 grassland sites. Our results indicate that elevational shifts in CN (carbon to nitrogen) ratios and herbivory depend on plant guild and season. CN ratios increased with altitude for grasses, but decreased for legumes and other forbs. In contrast to our hypotheses, extreme climatic events did not significantly affect CN ratios and herbivory. Thus, our study indicates that nutritional quality of plants and antagonistic interactions with insect herbivores are robust against seasonal climatic extremes. Across the three functional plant guilds, herbivory increased with nitrogen concentrations. Further, increased CN ratios indicate a reduction in nutritional plant quality with advancing season. Although our results revealed no direct effects of extreme climatic events, the opposing responses of plant guilds along elevation imply that competitive interactions within plant communities might change under future climates, with unknown consequences for plant-herbivore interactions and plant community composition.

## Introduction

Plants and herbivorous insects represent estimated 50% of all species and herbivory plays a major role in shaping plant community diversity and composition [1]. Climate change will globally increase temperatures and the frequency of extreme events [2], but the consequences for plant-herbivore interactions are little understood [3]. Climate change and extreme events can change the phenology and performance of plants and herbivorous insects [4], [5]. This might lead to phenological desynchronisation, increased or decreased herbivory, shifts in competitive strength within plant communities and altered population dynamics of plants and herbivores [3], [6]. To analyse the impact of climate change and extreme events on plant-herbivore interactions two different approaches have been realised in ecological research: First, studies of plant-herbivore interactions along elevational gradients have been used to forecast responses to changing temperature and precipitation patterns [7], [8]. Second, the simulation of extreme climatic events at single locations is a promising experimental approach to evaluate possible responses of plants and their interactions with insects [9], [10]. However, gradient and experimental approaches have been rarely combined, thereby limiting the conclusions about impacts of climate change either to gradual shifts or to the, in most cases ambient temperate, local climatic context [11]–[13]. However, the strongest effects of climate change are expected in mountainous and arctic ecosystems [14]. Therefore, a promising approach is the combination of manipulative climate experiments with elevational gradients.

In the European Alps, particularly at higher altitudes, climate change is already observable by a three times stronger temperature increase than the global-average 20^th^ century warming [15], shifts in the elevational distribution of plants and insects [16], [17] and heavier precipitation events in alpine regions, particularly during winter time [18], [19]. It is expected that the length of the winter season will further decrease, the fraction of liquid precipitation will increase and particularly at lower altitudes higher temperatures are predicted to accelerate snowmelt [20]. Therefore, future climate change scenarios predict either higher or lower snow cover depending on altitude and location [21]. In contrast, rainfall in summer is predicted to decrease and the frequency of drought events to increase across Europe [22].

Climatic conditions might affect chemical and physical plant defences against herbivores, CN (carbon to nitrogen) ratios of plants and thereby host plant quality and diet breadth of herbivores [23]–[26]. Low temperatures and a short growing season at higher altitudes have been proposed to increase plant N concentrations and to decrease concentrations of secondary defence compounds due to harsh environmental conditions and low herbivore pressure [8], [27]. Simulated drought can change leaf N concentrations and CN ratios [28], [29], but standardised drought experiments along climatic gradients also addressing the consequences for plant-animal interactions, are lacking [3], [30], [31].

Invertebrate herbivores might profit from climatically stressed plants due to increased leaf nitrogen concentrations [32] and decreased concentrations of secondary defence compounds [33]–[35]. In addition, climatic events such as advanced and delayed snowmelt can differently shift the phenology of plants and herbivores, thereby desynchronising interactions and altering herbivory [36], [37]. Generally, herbivore densities and herbivory are expected to be highest at the beginning of the growing season, when leaf N concentrations are highest, and to decrease until leaf senescence. However, compensatory feeding on plants with low leaf N concentration can result in increased herbivory [38].

At a plant community level, different plant guilds vary in CN ratios and leaf N concentrations. Legume forbs (thereafter legumes) have in contrast to non-legume forbs (thereafter forbs) and grasses lower CN ratios and higher leaf N concentrations, which can influence the preference of insect herbivores towards legumes and might result in guild specific differences directed by climate change. In this study we performed a replicated climate change experiment to investigate the effects of simulated extreme climatic events (advanced snowmelt, delayed snowmelt, extreme drought versus control plots) on plant-herbivore interactions along an elevational gradient in the Alps from 600–2000 m elevation. In each treatment we measured CN ratios and herbivory for representative plant species of the plant guilds grasses, legumes and forbs to test the following main predictions:

1. In control plots CN ratios and herbivory decrease with increasing elevation and differ between plant guilds.
2. Extreme drought hampers plant defense and increases herbivory particularly at low elevations with high herbivore pressure.
3. Advanced snowmelt leads to an earlier plant growth and reduces herbivory pressure by desynchronisation of plant and herbivore phenology.
4. Delayed snowmelt increases herbivory due to the availability of young plants with high N concentrations later in the season.
5. Effects of extreme events are more pronounced early in the season and in plant guilds with low plant N concentrations.

## Material and Methods

### Study region

The study was conducted in the Berchtesgaden National Park in the southeast of Germany and its surroundings (47°6′ N, 12°9′ E). The Berchtesgaden National Park is located in the Eastern Alps and is characterised by alpine meadows and mountains. The mountains are up to 2700 m a.s.l.. Average annual rainfall varies between 1500 and 2600 mm and average annual temperature ranges from −2°C to +7°C depending on the altitude. We thank Michael Vogel, Helmut Franz and the Berchtesgaden National Park for the permission to work in the national park and the owners of the study sites outside the national park for their allowance.

### Climate experiment

**Figure 1:**
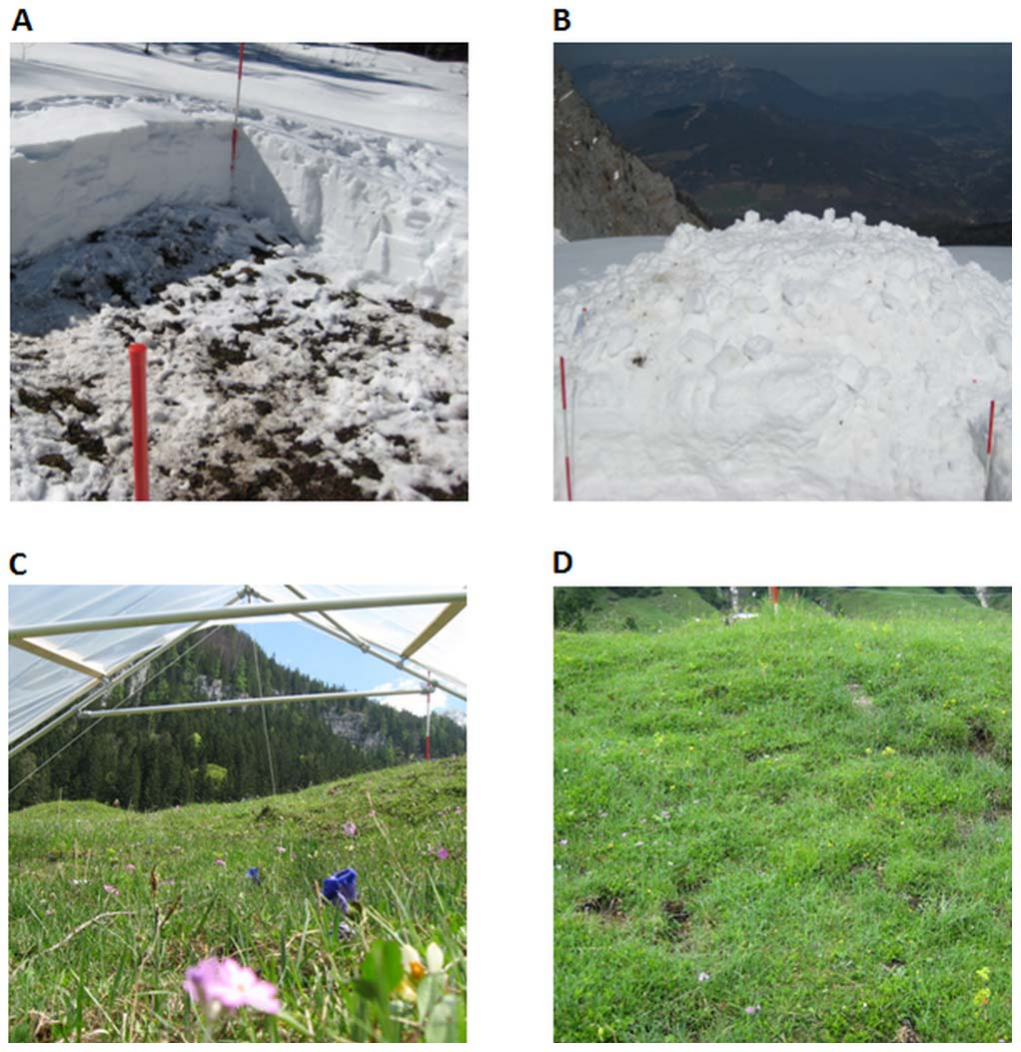
Experimental design with four treatments per study site. (A) advanced snowmelt, (B) delayed snowmelt, (C) drought treatment with rain-out shelter and (D) unmanipulated control. Each treatment plot measured 4×4 m and the distance between the plots was 1 m (Photos: A. Leingärtner and B. Hoiss).

Between February 20^th^ and April 8^th^ 2010 we selected 15 study sites along an elevational gradient (600 to 2000 m a.s.l.) to test whether winter/spring climate change can affect food quality and herbivore damage of alpine plants. We conducted four climate treatments in 4×4 m plots at each study site: (A) advanced snowmelt, (B) delayed snowmelt, (C) extreme drought and (D) control (Figure 1). To simulate advanced snowmelt we removed snow from a randomly chosen plot after the last snowfall in early spring until only a thin layer was left and shoveled the snow on an adjacent plot to increase snow cover and simulate a delayed snowmelt. The four plots were separated by 1 m wide corridors at each site. We removed a snow layer of 73±57 cm (range: 15–214 cm) from advanced snowmelt plots. Snow layer depth varied between the study sites depending on altitude, exposition and inclination. The snow layer at the delayed snowmelt plots, after snow shoveling, varied between 16 and 304 cm along the alpine gradient and was on average 111±84 cm. We defined the plots as snow-free when near-surface air temperatures reached more than +5°C on at least three consecutive days [39].

To simulate an extreme drought event we constructed 4×4 m rain-out shelters with aluminium tubes and cast-iron key clamps (B-One key clamps, Montfoort, the Netherlands) and covered them with a transparent plastic sheet (0.2 mm polyethylene, SPR 5, Hermann Meyer KG, Germany), which allowed nearly 90% penetration of photosynthetically active radiation (Figure 1). We set up the rain-out shelters between 26^th^ April and 24^th^ June on average four weeks after snowmelt. The drought period lasted 43±1 days to simulate a 1000-year extreme event for the study region based on data from the German Weather Service [9]. The rain-out shelters had a roof height of 125 cm at the highest point and had two open sides to avoid greenhouse effects and allow air exchange. Mean near-surface air temperature under the rain-out shelters was 14.5±2.1°C during the drought period and 14.4±2.2°C on the control plots, thus no significant differences between rain-out shelters and control plots existed (paired t-test: *t*_12_ = −0.5, *P* = 0.6).

### Abiotic factors

We measured near-surface air temperatures with temperature loggers (Thermochron iButtons DS1921G#F5, Maxim Integrated Products, Inc., Sunnyvale, CA, USA) in the centre of each of the four treatment plots in 2 h intervals at all 15 study sites. Temperature loggers in the snowmelt treatments measured the subnivean temperatures near soil surface until snow had melted. We installed rain collectors to measure the amount of rain that was excluded from the drought treatment. Mean rainfall over all study sites during the drought period was 379±71 l/m^2^ and the amount of rain did not show a directional change along the elevational gradient (simple regression, *F*_1,13_ = 1.1, *P* = 0.3). We monitored soil moisture during the drought period for each treatment separately with a portable soil moisture meter at 60 mm depth (Delta-T Devices type HH2 + ThetaProbe ML2x sensors, Cambridge, UK). We measured soil moisture on average on 4±1 days per treatment and site at five positions within the treatment plots.

### CN ratios and leaf herbivory

We collected leaf samples at each of the four treatments at three times during the growing season at an interval of three weeks, to measure leaf carbon to nitrogen (CN) ratios. Each time we collected three leaves from each of five individual plants per species and treatment. We took samples from 7±2 plant species per study site representing all three plant guilds (grasses, legumes, forbs) with the widest occurrence on the four plots per site. Supporting information for CN data of the three plant guilds along altitude is given in Table S1. We tried to sample only healthy green leaves, however especially for grasses phenological aging occurred during the season and mostly less green individuals were available at the end of the season. In total we took 751 leaf samples from 42 plant species to determine leaf CN ratios. Leaf samples were oven-dried for 48 h at 75°C and afterwards analysed with an elemental analyser (vario MICRO cube, Elementar Analysensysteme GmbH, Hanau, Germany).

In parallel, we recorded herbivore damage of the same plant species as used for the CN analyses at three week intervals. We randomly chose five individuals per plant species and treatment and estimated percentage leaf area loss from 0 to 100% for each leaf by visual inspection. In total we estimated herbivory of 25,013 leaves of the three plant guilds (grasses, legumes, forbs) (see supporting information for herbivory data in Table S1). The accuracy of our estimates was a priori evaluated by 30 randomly collected leaves. These removed leaves were transferred to millimetre paper and the exact amount of missing leaf area was measured. We did not sample insect herbivores and can mainly exclude megaherbivores. The monitored herbivory on single leaves are only a measure of insect herbivory as vertebrate herbivores consume larger plant parts. Furthermore, we installed fences around our treatment plots, when the study sites were grassed by cattle or sheep (7 out of 15). We did not observe high densities or impact of wild deer on the unfenced study sites.

### Statistical analysis

We calculated linear mixed effects models with type I sum of squares [40] following the default setting of the software R 2.15.1 for Windows [41] and suggestions by Crawley [42]. We first tested the effects of the explanatory variables in the model entry sequence altitude (continuous), treatment (4 categories), plant guild (3 categories), sampling time (3 categories) and their interactions on the response variables CN ratio and herbivory. Models with the response variable CN ratio followed the assumptions of normality and homoscedasticity, but the variable herbivory was arcsine square root transformed to meet the assumptions in the final models. Nonetheless, calculating the models without transformations gave very similar results. We used mean values for each plant guild (grasses, legumes, forbs) and treatment (advanced snowmelt, delayed snowmelt, extreme drought, control) per study site and sampling time to average the effect of single plant species. We used study site, treatment and plant guild as random effects to account for nesting and in order to correct for pseudoreplication.

In a second step we tested the effect of CN ratio, plant guild and the interaction between CN ratio and plant guild on herbivory and in a final step we calculated the effects of altitude, treatment and the interaction of altitude and treatment on soil moisture and the time of snowmelt. In these models we used study site as random effect to correct for pseudoreplication. All models were fitted with likelihood ratio tests [42]. Tukey HSD tests were used to calculate differences between categories of significant main effects [42].

## Results

### Snow and drought experiments

**Figure 2:**
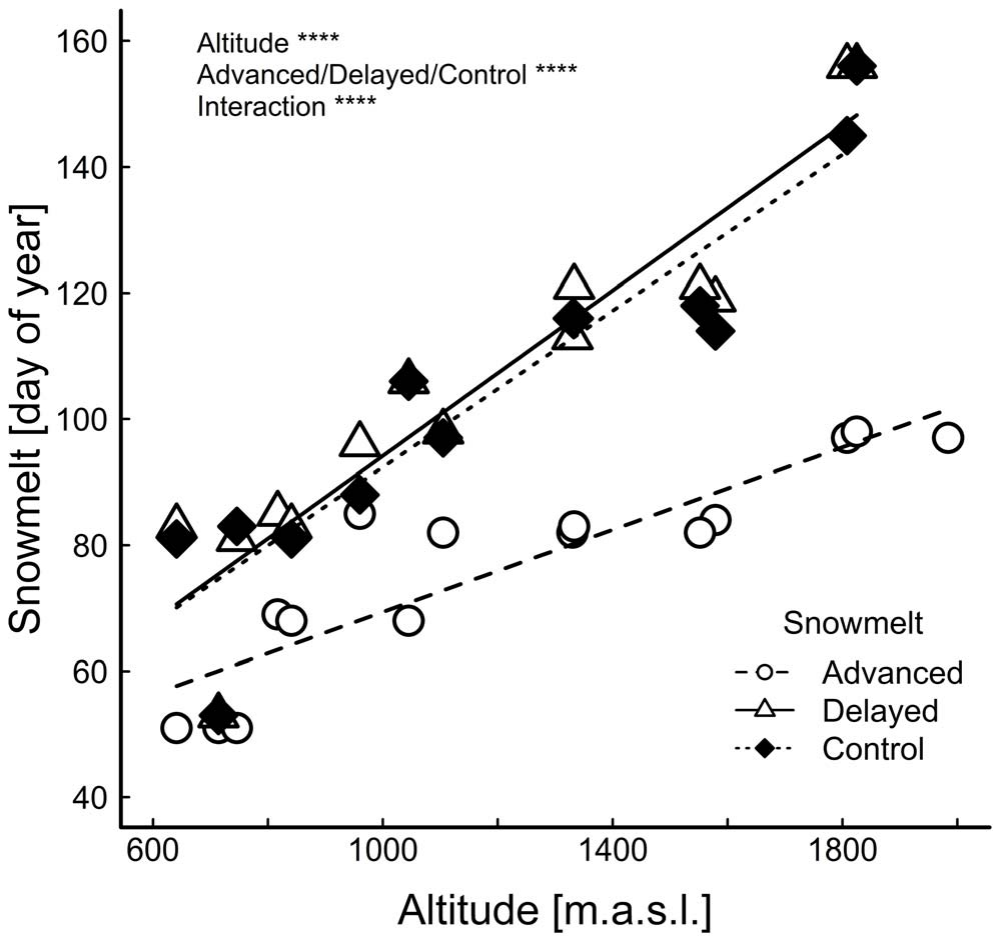
Final day of snowmelt for three different treatments (advanced snowmelt, delayed snowmelt, control) on 15 study sites in relation to altitude. **** *P*≤0.0001

**Figure 3:**
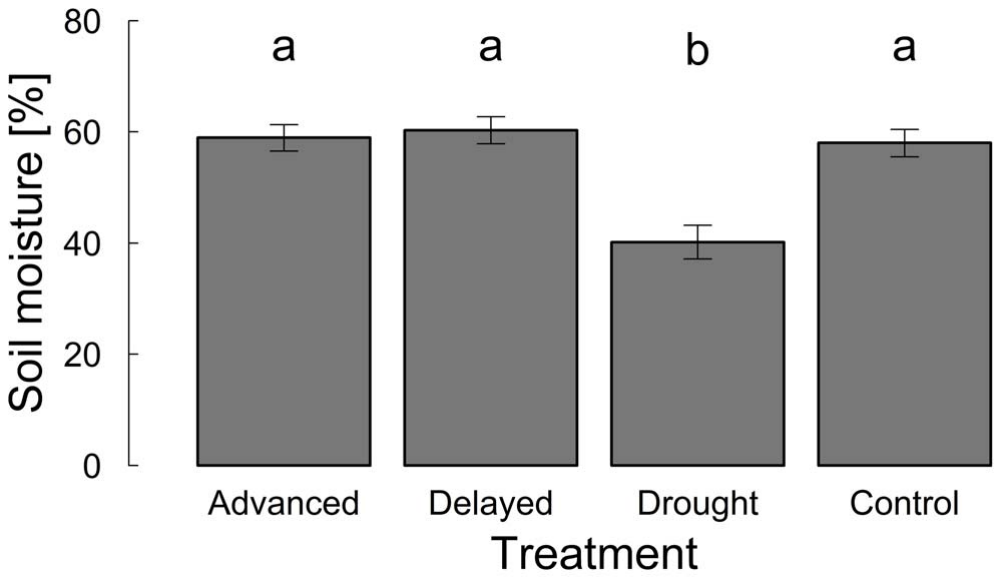
Soil moisture [% volume] during the extreme drought event on four treatments (advanced snowmelt, delayed snowmelt, extreme drought, control) (mean ± se). Letters indicate non-significant (a – a, *P*>0.05) and significant (a – b, *P*≤0.0001) differences between the treatments according to Tukey HSD post-hoc comparisons.

The time of complete snowmelt was successfully manipulated along the elevational gradient (Figure 2) without significantly increasing soil water availability (Figure 3). In control treatments snowmelt was finished on average over all study sites on 14^th^ April (104^th^ day of the year). On advanced plots snowmelt was finished on 18^th^ March (77^th^ day of the year) and on delayed plots on 15^th^ April (105^th^ day of the year) showing a mean advanced snowmelt of 27 days and a delayed snowmelt of one day. At higher altitudes treatment plots were naturally covered with more snow than at lower altitudes thus the time lag between advanced and control snowmelt was larger on higher plots compared with lower plots (Figure 2).

Soil moisture was significantly lower on drought plots during simulated extreme drought events compared to the other treatment plots (*F*_3,42_ = 26.5, *P*<0.0001, Figure 3). Drought plots had on average 40.2±3.0% soil moisture during the drought manipulation, whereas the other treatment plots had significantly higher soil moisture (control: 58.0±2.4%, advanced: 58.9±2.3%, delayed: 60.3±2.4%). The interaction of altitude and treatment was not significant, indicating a similar treatment effect along the elevational gradient.

### CN ratio

**Figure 4:**
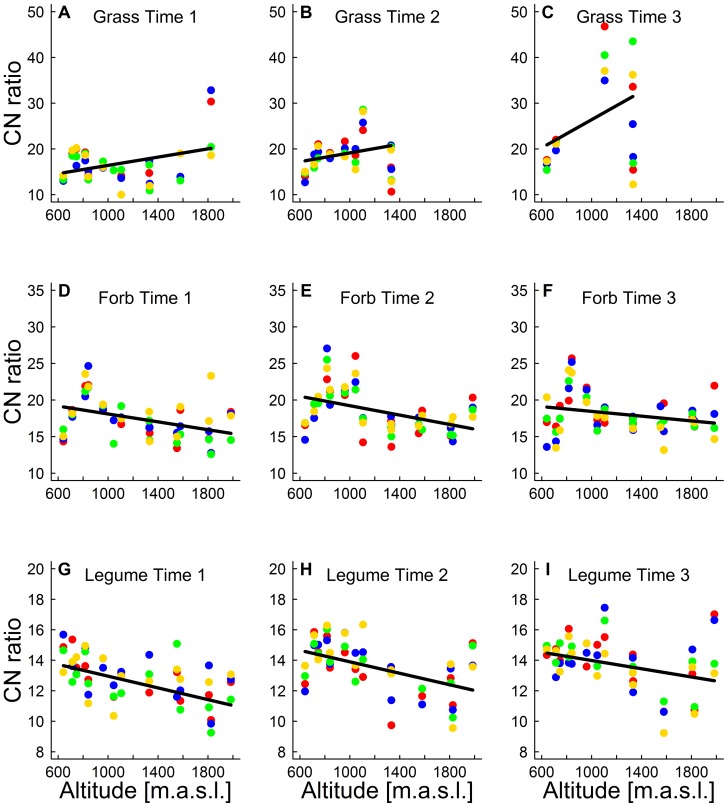
Effects of altitude, treatment and their interaction on CN ratio of three plant guilds (grasses, legumes, forbs) at three sampling times (A–I). CN data are presented as mean values per study site and treatment. Points symbolise the study sites and different colours represent the four treatments (blue: advanced snowmelt, green: delayed snowmelt, yellow: extreme drought, red: control). Black lines are based on the simplified model and show different slopes for CN ratio with altitude. Statistics see Table 1.

The CN ratio of plants was affected by the interaction of altitude, plant guild and sampling time (Table 1). Treatment (advanced snowmelt, delayed snowmelt, extreme drought, control) or any interaction with other variables had no significant effect on the CN ratio and was therefore removed as explanatory variable from the model (Table 1). The highly significant three-way interaction of altitude, plant guild and sampling time indicates that these three explanatory variables played an important role for the CN ratio of plants and depend in their effects on each other. Plant guilds (grasses, legumes, forbs) significantly differed in CN ratio (Figure 4). Legumes had the lowest CN ratio compared with forbs and grasses (*P*<0.0001), but forbs and grasses were not significantly different in their CN ratio (*P* = 0.1). CN ratios were lowest at the first sampling time, and increased three weeks (*P*<0.001) and six weeks later (*P* = 0.06). The significant interaction between the explanatory variables altitude and plant guild indicates contrasting shifts in CN ratios of the plant guilds along the elevational gradient (Table 1). CN ratios of grasses showed a slight increase with altitude whereas the CN ratios of forbs and legumes decreased with altitude (Figure 4). The significant interaction of plant guild and time indicates that the CN ratios of the three plant guilds changed differently during the growing season. We observed major increases of CN ratios of grasses over time, whereas CN ratios of legumes only slightly increased and CN ratios of forbs first increased and then decreased during the growing season.

**Table 1:** Mixed effects model statistics of the response variable CN ratio with the explanatory variables altitude, treatment, plant guild, sampling time and their interactions.

	numDF	denDF	*F*-value	*P*-value
**(Intercept)**	1	257	1114.37	<0.0001
**altitude**	1	13	2.11	0.17
**plant guild**	2	103	107.83	<0.0001
**sampling time**	2	257	31.46	<0.0001
**altitude:plant guild**	2	103	20.23	<0.0001
**altitude:sampling time**	2	257	0.57	0.57
**plant guild:sampling time**	4	257	38.03	<0.0001
**altitude:plant guild:sampling time**	4	257	4.53	0.002

The explanatory variable treatment was removed from the model as it was neither significant as single variable nor in the interactions. The final model is presented.

### Herbivory

**Figure 5:**
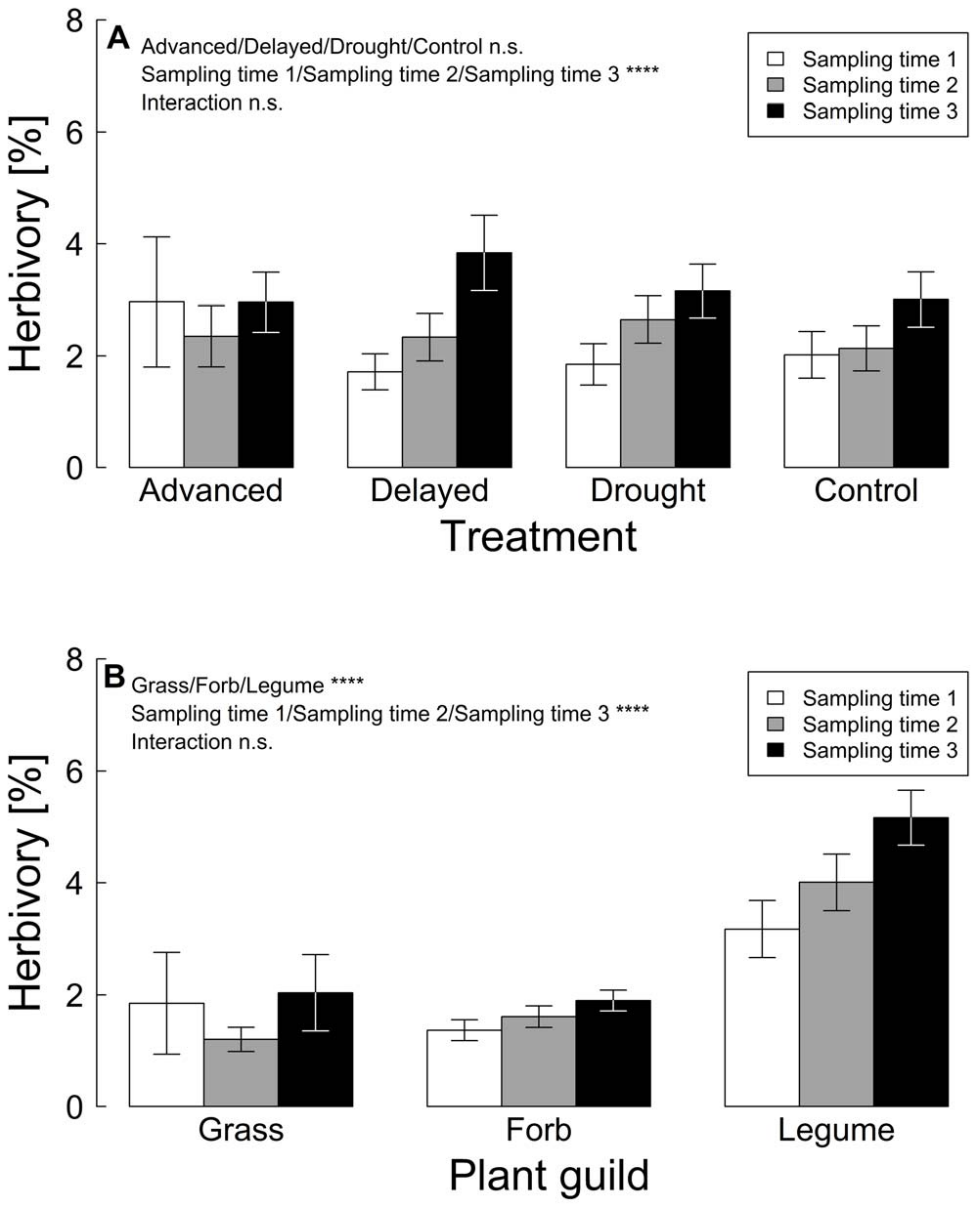
Herbivory [%] as a function of (A) treatment (advanced snowmelt, delayed snowmelt, extreme drought, control) and (B) plant guild (grasses, legumes, forbs) at three sampling times (mean ± se). **** *P*≤0.0001, n.s. *P*>0.1. Statistics see Table 2.

Herbivory was affected by the interaction of altitude, plant guild and sampling time (Table 2). Treatment and any interaction with treatment had no effect on herbivory (Figure 5A), therefore we removed the explanatory variable treatment and the interactions with treatment from the final model. Grasses, legumes and forbs had different herbivory which also changed with sampling time (Figure 5B). Legumes had a significantly higher herbivory compared to grasses and forbs (*P*<0.001), while grasses and forbs were not different in their herbivory (*P* = 0.9). At the first sampling time the herbivory was lowest but not significantly different to the herbivory three weeks later (*P* = 0.14). Six weeks later herbivory further increased, leading to significant differences between sampling times (Time 1 – Time 3: p<0.0001, Time 2 – Time 3: *P* = 0.02). The three-way interaction of altitude with plant guild and sampling time did not reveal clear overall elevational patterns in herbivory.

**Table 2:** Mixed effects model statistics of the response variable herbivory with the explanatory variables altitude, treatment, plant guild, sampling time and their interactions.

	numDF	denDF	*F*-value	*P*-value
**(Intercept)**	1	266	273.70	<0.0001
**altitude**	1	13	0.33	0.58
**plant guild**	2	103	41.90	<0.0001
**sampling time**	2	266	12.91	<0.0001
**altitude:plant guild**	2	103	1.14	0.32
**altitude:sampling time**	2	266	2.77	0.06
**plant guild:sampling time**	4	266	1.25	0.29
**altitude:plant guild:sampling time**	4	266	2.89	0.02

The explanatory variable treatment was removed from the model as it was neither significant as single variable nor in the interactions. Herbivory was arcsine square root transformed. The final model is presented.

### CN ratio - herbivory relationship

**Figure 6:**
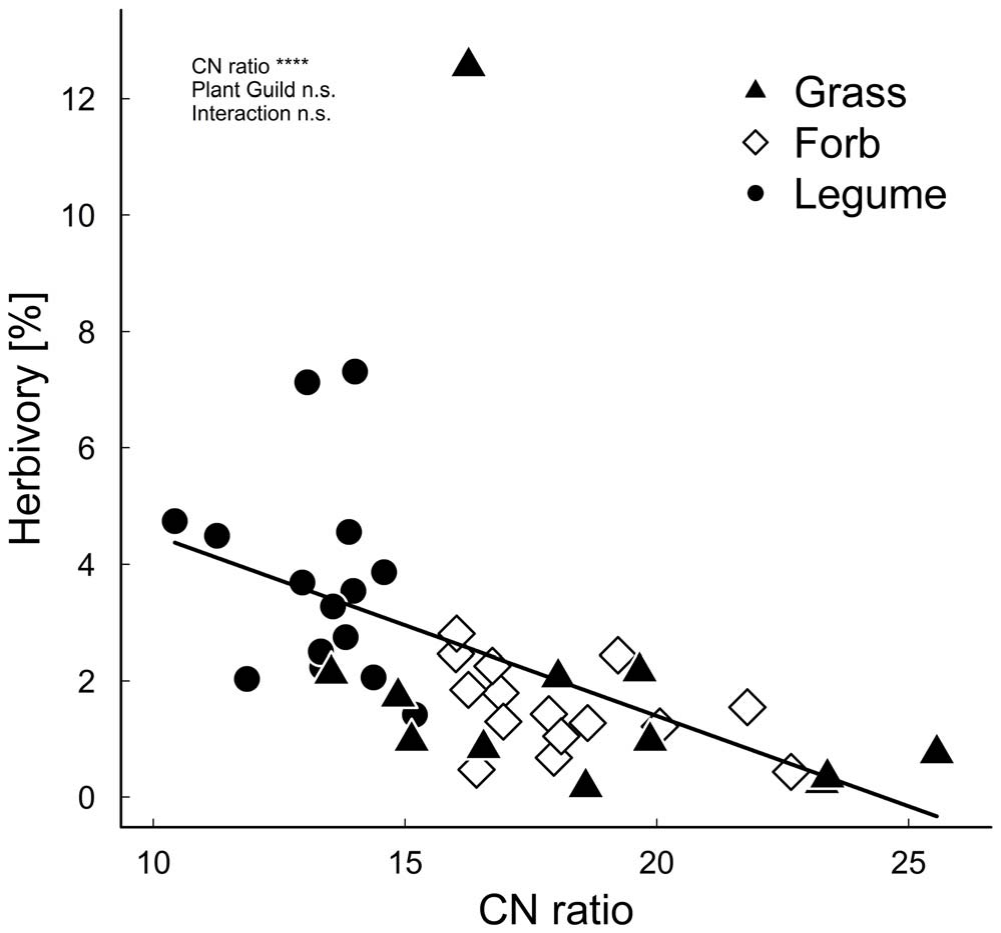
Relation between herbivory [%] and CN ratio of three plant guilds (grasses, legumes, forbs). The black line shows the significant relation between herbivory and CN ratio. **** *P*≤0.0001, n.s. *P*>0.1.

The CN ratio significantly affected herbivory (Figure 6), in that the more leaf nitrogen the plants had (low CN ratio) the higher was the herbivory of the plants. We found no interaction between CN ratio and plant guild suggesting identical slopes for the plant guilds. The explanatory variable plant guild did not explain further variation in herbivory in addition to the CN ratio.

## Discussion

Our results indicate that elevational shifts in CN ratios and herbivory depend on plant guild and season. Thus, the three plant guilds differed in CN ratios and herbivory and responded differently to altitude. In contrast to our predictions, extreme climatic events did neither affect CN ratios nor herbivory, suggesting that nutritional quality of plants and antagonistic interactions with insect herbivores are robust against seasonal climatic extremes. Across the three plant guilds, herbivory was highest when CN ratios were lowest, demonstrating that high N content is related to increased plant damage by herbivores.

### CN ratio

We expected that advanced snowmelt and drought would result in lower CN ratios compared to delayed snowmelt and control plots, particularly early in the season. However, we found no treatment effects on CN ratios. Other experimental studies that manipulated snow depth at multiple sites along a climatic gradient are rare and two climate experiments in arctic environments gave ambiguous results for snow cover manipulations [23], [43]. By replicating the climate experiment at multiple sites we can exclude that contrasting temperature conditions at low or high altitudes affected the response of plant CN ratios to snowmelt manipulations. Nevertheless, the advance in snowmelt increased significantly along the altitudinal gradient compared to control plots and the effects on flower phenology were more pronounced at higher altitudes shown in a recent study at the same study sites [44]. Therefore, removing the snow at higher or lower altitude can have a very different meaning and effect. However, the CN ratio showed no significant interaction between treatment and altitude indicating that CN ratios were robust to advanced snowmelt, despite changing treatment intensity. However, responses to manipulations may occur at different time scales and plant species might react with time delay to changing environmental conditions [45].

The extreme drought treatment in our study did not change plant CN ratios despite the simulation of a 1000 years drought event. We predicted lower CN ratios, because decreased soil moisture can result in slowed growth and higher concentration of plant leaf minerals [46]. Importantly, in our experiment the drought simulation did not increase temperatures on the plots and therefore we could independently analyse the response of plants to drought events. Thus, significant changes in CN ratios in other studies that simulated higher temperatures and drought in combination [29], might be more related to temperature increases than reduced soil moisture. We could not control for air humidity, thus plant species might have experienced higher air humidity during the simulated drought event than under natural extreme drought conditions, but this is also the case for other climate experiments. Another explanation why we did not find any patterns might be that the drought simulation was not strong enough. Even though we reduced soil-moisture significantly, the remaining water content of 40% might be still too high to represent a severe drought for most alpine plants [47].

Although we found no effects of drought, climatic conditions along the elevational gradient affected CN ratios and herbivory in concert with plant guild specific responses and seasonal shifts. Nitrogen fixing legumes had the lowest leaf CN ratios compared with grasses and forbs. Within all plant guilds, leaf CN ratios increased over time, indicating a decrease of leaf nitrogen concentration during the growing season, which adds to related results in woody plants [37], [48]. Mechanisms explaining seasonal decreases in leaf nutrient concentrations are the accumulation of carbon during the growing season, which operates as a dilution effect, and the recovery of nutrients from leaves before leaf senescence [49]. The significant interaction with plant guild and sampling time indicates that altitude influenced the CN ratios of grasses, legumes and forbs in different directions. Legumes and forbs had at all three sampling times lower CN ratios at higher altitudes, but within grass species CN ratios increased with altitude. However, owing to the lack of grass data at the end of the season, the interpretation of CN ratios of grass species remains speculative. During the sampling periods we tried to collect only healthy green leaves, however phenological aging of leaves may have caused the increase in CN ratios of grass species later in the season.

### Herbivory

In contrast to our predictions insect herbivory was not affected by simulated climate change, presumably because nutritional quality of plants remained unchanged during the sampling period. However, our data imply that herbivores are strongly driven by plant leaf nutrients. The CN ratios were a very good predictor to explain herbivory and herbivores preferred food plants with a lower CN ratio and therefore higher N content in the leaves. Related to this, the leaf nutrient contents of the different plant guilds (grasses, legumes, forbs) influenced herbivory. Legumes had on average the highest herbivory compared with grasses and forbs, which can be explained by low leaf CN ratios and therefore high N contents in legumes. Additionally, we found changes in herbivory during the growing season. Herbivory of all plant guilds increased with time, thereby the increase varied on average between 0.2% for grass species, 0.5% for forb species and 2% for legume species. However, herbivory differed between species ranging from 0.03% in *Campanula rotundifolia* to 7.8% in *Vicia cracca* with presumably different effects on plant performance. An increase in herbivory despite decreasing nutritional plant quality later in the season can be explained by an increased consumption rate of either insect larvae of advanced instar stages or abundance peaks of herbivores later in the season. While herbivory changed with season, we found no overall effect of altitude on herbivory, in contrast to our predictions. However, herbivory varied between plant guilds and sampling time along the elevational gradient. Other studies suggest that the abundance of herbivorous insects and leaf herbivory decrease with increasing elevation [8], [50], although increasing food plant quality might increase herbivory at higher elevations [27]. Thus, the lower CN ratios of legumes and forbs at higher altitudes with potentially increased herbivory might be counteracted by lower herbivory on grasses and elevational declines of herbivore populations, and therefore no overall pattern in insect herbivory along the elevational gradient occurred. However, as we did not measure herbivore abundances at our study sites, differences in herbivore abundances between sites can also explain the missing pattern along elevation.

In conclusion, our results indicate that shifts in the snowmelt date and extreme drought events in the German Alps had no strong effect on food plant quality and insect herbivory. Although mean effects of climate manipulations on plant species and herbivores were not significant in our experiment, the opposing responses of CN ratios in forb (legume and non-legume) and grass plant guilds to altitude imply that competitive interactions within plant communities might change under future warmer climates, with unknown consequences for plant-herbivore interactions. Therefore, we recommend long-term experiments simulating multiple extreme climatic events along climatic elevational or latitudinal gradients to reveal the complex dynamics and potential risks of future climate change for biotic interactions and ecosystem stability.

## Supporting Information

Table S1 **CN ratios and herbivory of three plant guilds (grasses, forbs, legumes) along altitude.**
(DOCX)
