# Supplementary material for: Combined Effects of Extreme Climatic Events and Elevation on Nutritional Quality and Herbivory of Alpine Plants
Source: PLoS One. 2014 Apr 4;9(4):e93881. doi: 10.1371/journal.pone.0093881 (PMC3976348; doi:10.1371/journal.pone.0093881)
Supplement: Table S1 — CN ratios and herbivory of three plant guilds (grasses, forbs, legumes) along altitude. (DOCX) [file pone.0093881.s001.docx]

**Supporting Information**

**Table S1. CN ratios and herbivory of three plant guilds (grasses, forbs, legumes) along altitude**

| **altitude [m.a.s.l.]** | **plant_guild** | **CNratio** | **herbivory** |
| --- | --- | --- | --- |
| 641 | grass | 14.78 | 1.73 |
| 641 | forb | 16.11 | 1.97 |
| 641 | legume | 13.95 | 7.52 |
| 714 | grass | 19.18 | 2.16 |
| 714 | forb | 17.27 | 1.55 |
| 714 | legume | 14.42 | 2.17 |
| 746 | grass | 19.24 | 0.97 |
| 746 | forb | 18.79 | 1.59 |
| 746 | legume | 14.01 | 4.56 |
| 817 | grass | 18.59 | 0.17 |
| 817 | forb | 22.92 | 0.53 |
| 817 | legume | 15.07 | 1.42 |
| 841 | grass | 16.43 | 0.97 |
| 841 | forb | 22.60 | 1.67 |
| 841 | legume | 13.39 | 2.75 |
| 960 | grass | 18.04 | 2.06 |
| 960 | forb | 20.31 | 1.42 |
| 960 | legume | 14.64 | 3.86 |
| 1045 | grass | 16.97 | 0.84 |
| 1045 | forb | 19.23 | 2.72 |
| 1045 | legume | 13.00 | 5.08 |
| 1105 | grass | 26.60 | 0.20 |
| 1105 | forb | 17.45 | 1.05 |
| 1105 | legume | 14.42 | 3.23 |
| 1330 | grass | 24.04 | 0.35 |
| 1330 | forb | 17.08 | 1.98 |
| 1330 | legume | 13.40 | 4.77 |
| 1333 | grass | 13.53 | 2.12 |
| 1333 | forb | 15.68 | 3.05 |
| 1333 | legume | 11.86 | 2.03 |
| 1552 | forb | 16.08 | 3.13 |
| 1552 | legume | 13.33 | 2.50 |
| 1579 | grass | 16.26 | 12.55 |
| 1579 | forb | 16.95 | 1.30 |
| 1579 | legume | 11.23 | 4.53 |
| 1808 | forb | 16.42 | 0.47 |
| 1808 | legume | 13.06 | 7.13 |
| 1825 | grass | 25.56 | 0.75 |
| 1825 | forb | 15.89 | 1.32 |
| 1825 | legume | 10.30 | 4.50 |
| 1984 | forb | 17.96 | 0.68 |
| 1984 | legume | 13.98 | 3.54 |
